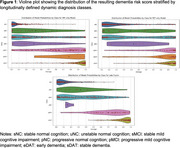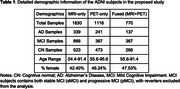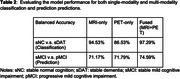# Multi‐modal Neuroimaging Based Dementia Risk Score for Early Detection of Future Risk of Dementia Onset for Alzheimer's Disease

**DOI:** 10.1002/alz70862_110136

**Published:** 2025-12-23

**Authors:** Swapnil Singh, Marc D. Rudolph, Trey R. Bateman, Timothy M. Hughes, Kiran K. Solingapuram Sai, Suzanne Craft, Metin Nafi Gurcan, Karteek Popuri, Mirza Faisal Beg, Liqing Zhang, Da Ma

**Affiliations:** ^1^ Virginia Tech, Blacksburg, VA USA; ^2^ Wake Forest University School of Medicine, Winston‐Salem, NC USA; ^3^ Memorial University Of Newfoundland, St. John’s, NF Canada; ^4^ Simon Fraser University, Burnaby, BC Canada

## Abstract

**Background:**

Alzheimer’s disease (AD) is defined by multi‐domain biomarkers according to the revised classification framework. Machine learning models may benefit from an effective combination of multiple modalities. This study aims to incorporate multi‐modal neuroimaging data (T1‐MRI and amyloid‐PET) to improve the predictive power of deep learning models, capturing both the amyloid (A) and atrophy (N) patterns in the brain to derive dementia risk score (DRS) and prediction risk of future progression of dementia at the early stage of the AD.

**Method:**

We used the multi‐modal neuroimaging data from the ADNI 1,2 and GO datasets (Table 1). The CN and AD subjects were used to train a classification model through 5‐fold cross‐validation to learn AD‐related neuroimaging features and derive the dementia risk scores. The derived models were then applied to subjects who were diagnosed as MCI at their baseline measurements to predict the future risk of progression to dementia. Both T1‐MRI and Amyloid‐PET data were rigid‐registered to the MNI space and skull‐stripped. ResNet50 models with initial model weights pre‐trained from MedicalNet were fine‐tuned to train classification tasks for MRI and PET independently. The resulting single‐modal DRS was then averaged to achieve a fused multi‐modal DR. The multi‐modal DRS was then used to infer the final prediction for future dementia onset. Predictive performance was evaluated via balanced accuracy and AUC.

**Result:**

When classifying AD/CN, the balanced accuracy is 94.53% for the MRI‐only model, 86.53% for the PET‐only model; and 97.29% for the fused model. For predicting future MCI progression, the balance accuracy was 71.17**%** for MRI‐only model, 71.79% for the PET‐only model, and 74.59% for the fused model (Table 2, Figure 1).

**Conclusion:**

This study underscores the potential of leveraging multi‐modal deep learning models toward improving accuracy in AD prediction and tracking progression. Results demonstrated complementary strengths of MRI and PET. The reduced performance on pMCI prediction indicates room for improvement with further fine‐tuning process, more advanced multi‐modal fusion strategy, as well as the best modality (e.g. FDG‐PET and tau‐PET). Future plans involve multi‐modal fusion at an early stage of the model and further evaluate model generalizability using independent datasets such as NACC data.